# Bereavement practices employed by hospitals and medical practitioners toward attending funeral of patients: A systematic review

**DOI:** 10.1097/MD.0000000000016692

**Published:** 2019-09-06

**Authors:** Kwangtaek Kim, Leonid Churilov, Andrew Huang, Laurence Weinberg

**Affiliations:** a Department of Anaesthesia, Austin Health; b Department of Medicine (Austin Health), Melbourne Medical School, The University of Melbourne, Heidelberg, Australia.

**Keywords:** funeral rites, grief, physician–patient relationship

## Abstract

**Objectives:**

To ascertain bereavement practices offered by hospitals and medical practitioners (MPs), factors that influence the likelihood of MPs’ involvement in funeral attendance, the benefits and barriers to attendance to a patient's funeral as perceived by MPs and the rate of attendance to patients’ funeral by MPs.

**Design:**

MEDLINE (Ovid), Embase, PubMed, and Google Scholar were searched with a systematic search structure for randomized controlled trials, comparative observational studies, case series, cross-sectional studies, editorials, and letters. The search was limited to English only. The study was registered with Prospero (Registration Number: CRD42018095368).

**Results:**

A total of 381 articles were identified with 46 articles meeting the inclusion criteria. Of the 46, 16 were editorials and 12 were letters. Eighteen were cross-sectional studies conducted in the United States, Canada, Australia, Israel, and Ireland. Year of publication ranged from 1990 to 2017. Of these, 12 were quantitative, 3 were qualitative, and 3 were mixed-method studies. Two of the cross-sectional studies involved family members of deceased patients while others involved MPs. Bereavement practices offered by hospitals included memorial services, letters, and services provided by bereavement coordinators. Bereavement practices employed by MPs included answering or making phone calls, attending family meetings, and sending condolence letters. MPs’ attendance at a patient's funeral was influenced by MPs’ gender, age years of experience the medical specialty. Perceived benefits of MPs’ attendance at a patient's funeral included providing support to the family, extending the professional relationship, illustrating respect to the patient and the family, resolving guilt and personal growth. Barriers to the attendance included a lack of time, blurring of professional boundaries, personal discomfort with death, emotional arousal, and discouragement by colleagues. General practice had an attendance rate of 71%. Attendance rates for palliative care, oncology, and psychiatrists ranged from 63% to 81%, 7.1% to 67%, and 15% to 67%, respectively. Intensivists had an attendance rate of 22%.

**Conclusion:**

Several bereavement practices are provided by hospitals and MPs. Funeral attendance is an uncommon bereavement practice. MPs’ attitudes toward attending a patient's funeral are understudied in many specialties. Patient factors that influence MPs’ participation in bereavement practices are poorly understood.

## Abstract 2

Supplemental Digital Content is available in the text

## 1 Introduction

Medical practitioners (MPs) encounter the death of patients early in their training, and it can be a confronting and traumatic experience.^[1,2]^ Hospitals have attempted to minimize the stress of clinicians and patients’ families by facilitating commemoration services,^[3–5]^ writing condolence letters,^[6]^ and making phone calls to the deceased patient's family.^[6]^ Doctors attending a patient's funeral appears to be an uncommon practice.^[1,7–21]^

The motivation of clinicians who attend a patient's funeral (including the potential benefits) appears to be under-researched. While the literature outlines some of the reasons for funeral attendance,^[7–14,16–21]^ the rate, benefit, and barriers were limited to certain specialties.

Therefore, we implemented a systematic review to explore bereavement practices including funeral attendance employed by hospitals and MPs along with factors that influence their involvement in such practices.

## 2 Methods

This systematic review was completed according to the Cochrane guidelines.^[22]^ The study was registered with Prospero (Registration Number: CRD42018095368). As this study is a systematic review that does not involve direct patient care, Ethics approval and informed consent are not required.

### 2.1 Inclusion and exclusion criteria

The search included all study types ranging from randomized controlled trials, comparative observational studies, case series, cross-sectional studies, editorials, and letters that describe doctors’ attendance at a patient's funeral. We excluded studies relating to allied health professionals. The review focused on identified studies exploring the rate, reason, outcome, benefits, or barriers related to MPs attending a patient's funeral, and other types of bereavement practices employed by the hospital or a doctor. The search was confined to English language only.

Primary outcomes:

1. Types of bereavement practices offered to families by hospitals and MPs
2. Factors influencing an MP's attendance at a patient's funeral
3. Rate of funeral attendance by MPs

Secondary outcomes:

1. Prevalence of each bereavement practice
2. Factors influencing an MP's participation in a bereavement practice
3. Factors prompting an MP to attend a patient's funeral
4. Benefits of an MP's attendance at a patient's funeral
5. Barriers to an MP's attendance at a patient's funeral
6. Difference in the rate of funeral attendance in different medical specialties

### 2.2 Search strategy

**Figure 1:**
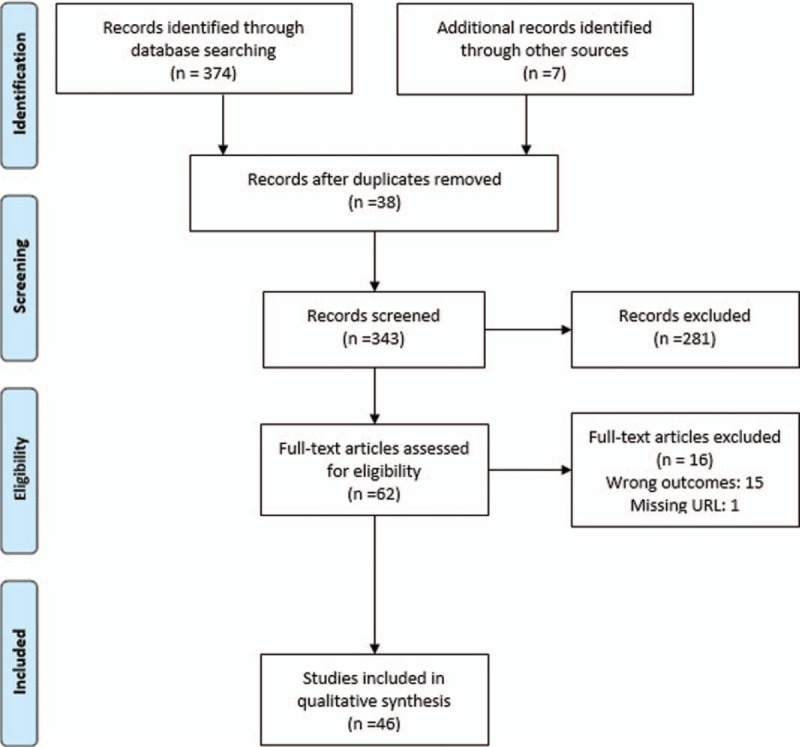
Prisma flow diagram.

Medline (Ovid), Embase, PubMed, and Google Scholar were used to search the literature in March 2018 without limiting for publication year. Full search histories of Medline (Ovid) and Embase, including the Medical Subject Headings (MeSH) and keywords, are listed in Appendix 1. Additional articles were identified from the bibliography of relevant articles. Two authors, KK and LW, screened the titles and abstracts to determine eligibility, completing the process independently and in a blinded manner. The studies were categorized as either included, excluded, unclear, or duplicate. Any disputes were referred to a 3rd author (AH) for review. A PRISMA flow diagram for the literature search is supplied as a guideline flow diagram (Fig. 1).

### 2.3 Study selection

A total of 381 articles were identified using our search strategy, including 46 articles from Medline (Ovid), 310 from Embase, 10 from PubMed, and 8 from Google Scholar. Seven additional articles were derived from bibliographies of relevant articles. Thirty-eight duplicates were excluded. The remaining 343 articles were screened according to journal type, title, and abstract. Based on screening, 281 publications were removed. The remaining 62 papers were read in full text, and 16 articles were excluded after the application of the selection criteria. Articles excluded described MPs’ personal experiences after a patient's death without explicit discussion about funeral attendance, or illustrated the practical aspects of post-death management, such as writing a death certificate. In total, 46 articles were included in this review.

### 2.4 Risk of bias and quality assessment

Two authors (KK, LW) independently assessed the risk of bias, and a 3rd author (AH) resolved any disputes. The risk of bias in the quantitative and mixed-method cross-sectional studies was assessed using Critical Appraisal of a Survey from the Centre for Evidence-Based Management.^[23]^ The qualitative cross-sectional studies were assessed for risk of bias with the Critical Appraisal Skills Programme Qualitative Checklist.^[24]^ Selective outcome reporting bias was evaluated based on a comparison of the reported results and the outcomes of the included studies. A funnel plot for the formal assessment of publication bias was not included, since the homogenous effect size across the studies was not relevant to this review.

### 2.5 Study characteristics

All studies were written in English, and the publication dates ranged from 1985 to 2017. Sixteen articles were editorials,^[4,5,7–9,12,17–21,25–29]^ and 12 were letters.^[1,10,11,13–16,30–34]^ The remaining 18 publications were cross-sectional studies^[2,3,6,35–49]^: 12 of them yielded quantitative data,^[35,37–41,43–48]^ 3 produced qualitative data,^[3,6,36]^ and 3 used mixed-method research designs.^[2,42,49]^

The majority of the cross-sectional studies were conducted in the United States (n = 11).^[2,32,40–47,49]^ Other countries included Australia (n = 3),^[6,35,36]^ Canada (n = 2),^[3,37]^ Israel (n = 1),^[39]^ and Ireland (n = 1).^[48]^ The number of participants in the cross-sectional studies ranged from 7^[36]^ to 535.^[37]^ Two of the studies^[3,49]^ involved the families of the deceased patients only, while 16 involved MPs only.^[2,6,35–48]^ The context of the studies that involved families was a tertiary pediatric Intensive Care Unit^[3]^ and a family practice.^[49]^ Of the studies that included MPs, most included a single specialty: emergency physicians,^[41]^ oncologists,^[39]^ pediatricians or pediatric trainees,^[2,40,42–45]^ palliative care physicians,^[36]^ and psychiatrists.^[48]^ Four of the studies purposefully sampled 2 specialties^[6,37,46,47]^ and 2 of the studies pertained to MPs, irrespective of their specialty.^[35,38]^

The cross-sectional observational studies varied in terms of methodology. Six of them employed an online survey,^[35,39,42,43,45,47]^ 8 of them utilized hard-copy questionnaires,^[2,38,40,41,44,46,48,49]^ and 3 conducted face-to-face interviews.^[3,6,36]^ One study used both an online survey and a hard copy questionnaire.^[37]^ MP's anecdotal experiences and personal beliefs about bereavement practices, and attendance at a patient's funeral was investigated in almost all of these studies. A single study used 8 imaginary clinical scenarios to examine 3 factors potentially influencing a clinician's attendance at patients’ funerals.^[43]^ Details of cross-sectional studies included in this study are summarized in Table 1.

**Table 1:**
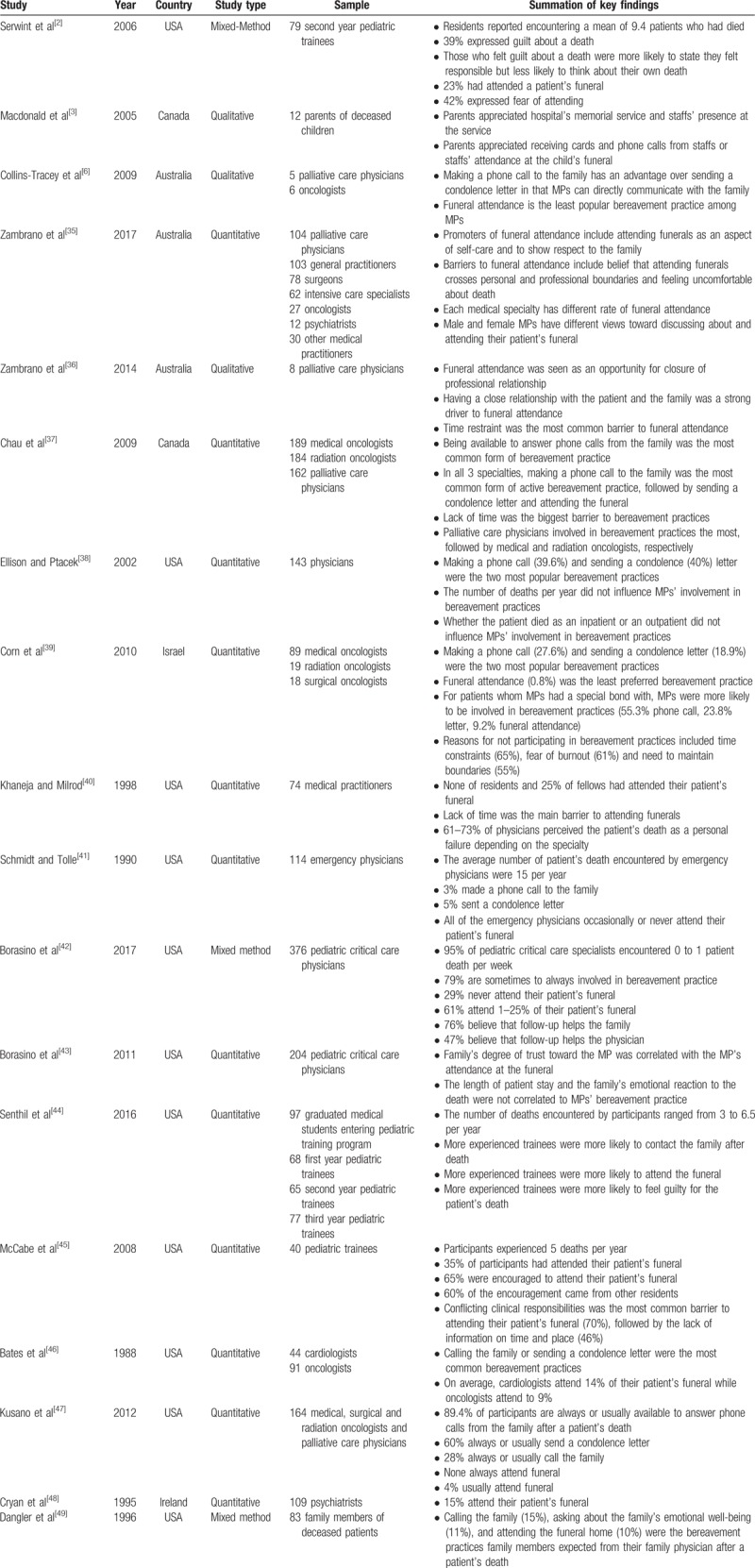
Summary of cross-sectional studies exploring bereavement practices of hospitals and medical practitioners toward attending funeral of patients.

### 2.6 Synthesis of results

Due to the significant heterogeneity of the methodology and study characteristics of the selected papers, a meta-analysis was inappropriate. Primary and secondary outcomes will be used as subheadings to structure the extracted data. Narrative synthesis will be used as appropriate to outline the study characteristics, methodology, main findings, and limitations.

### 2.7 Bereavement practices offered by hospitals and MPs

Traditionally, hospitals offered bereavement services to the families of the deceased patients to aid the healing process.^[3,5]^ Examples included facilitating memorial services to commemorate loved ones^[3–5]^ and providing condolence letter writing templates for healthcare professionals to use.^[6]^ Some hospitals even employed bereavement service coordinators who could deliver specific bereavement services to the relatives of the deceased patients.^[6]^

The MPs employed a diversity of bereavement practices,^[37,38,42,47]^ which have been categorized into passive and active practices. Passive practices included answering phone calls from the family, and attending requested family meetings.^[37,47]^ Active practices included sending condolence letters, making phone calls, and attending the patient's funeral or wake.^[37,47]^

Table 2 summarizes the types of bereavement practices commonly employed by MPs. While being available to answer phone calls from the patient's family is a common bereavement practice, Collin-Tracey et al^[6]^ speculated whether families interpreted the patient's death as the end of the professional relationship. Of active bereavement practices, sending a condolence letter and making a phone call to the family were the most commonly adopted. Block^[27]^ suggested that sending a condolence letter was preferable to making a phone call to the family, as the family can read the letter over and over. In contrast, Dangler et al^[49]^ reported that families usually expected to receive a phone call from the treating doctor. In addition, Collins-Tracey et al proposed that doctors preferred phone calls over condolence letters because they felt more comfortable talking to relatives than writing a letter.

**Table 2:**
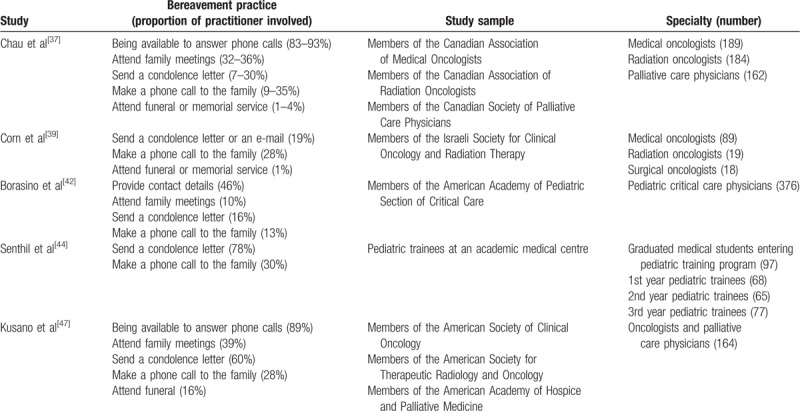
Bereavement practices commonly employed by medical practitioners.

### 2.8 Factors that influence participation in bereavement practices

Several factors influenced an MP's participation in bereavement practices. Being a female (odds ratio 2.2, *P* < .001), access to the bereavement program (odds ratio 2.34, *P* < .001) and an increasing number of patient deaths per month (odds ratio 1.03, *P* = .03) were associated with a higher likelihood of involvement in bereavement practices.^[37]^ Borasino et al^[42]^ also concluded that female pediatric critical care specialists were more likely to be involved in bereavement practices (odds ratio 2.1, 95% confidence interval [CI] 1.1–4.4). In addition, palliative care specialists were more likely to provide bereavement services, compared to oncologists.^[37]^ About 34.8% of palliative care specialists reported that they always or usually make a phone call to the family, as opposed to medical oncologists (21.3%) and radiation oncologists (9.3%) (*P* < .001). In addition, palliative care specialists were more likely to send a condolence letter (29.5%) than medical (18.2%) or radiation oncologists (7.1%) (*P* < .001). Of passive bereavement practices, palliative care physicians were more likely to initiate a family meeting than medical and radiation oncologists (13.4%, 6.4%, and 3.3%, respectively, *P* = .002).

### 2.9 Funeral attendance as a bereavement practice

The MPs’ views on attending their patients’ funerals were highly variable.^[6]^ Many MPs shared their experience of attending the funerals of their patients and encouraged others to attend.^[1,7–21,26,28,29]^ Nonetheless, quantitative studies clearly showed that funeral attendance is the least preferred bereavement practice.^[37–39,41,42,47]^ Moreover, although the majority of families greatly appreciated the attendance of an MP, they did not expect the practitioner, including the patient's family physician, to be present at the funeral.^[49]^

### 2.10 Factors that influence attendance at patients’ funerals

Despite a relative paucity of research, previous studies identified several factors associated with the rate of funeral attendance and have suggested potential benefits of, and barriers to attendance. Factors that influence MPs’ attendance at their patient's funeral are summarized in Table 3.

**Table 3:**
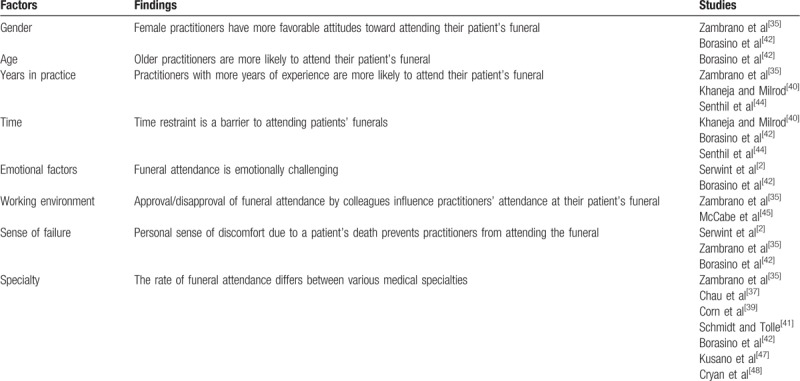
Factors that influence medical practitioners’ attendance a patient's funeral.

Female practitioners have more favorable attitudes toward funeral attendance. Zambrano et al^[35]^ suggested that among Australian MPs, female practitioners were more likely to discuss funeral attendance with their colleagues (*P* = .012), patients or families (*P* = .008). They were also more likely to agree that discussions about funeral attendance should take place during training (*P* = .003).^[35]^ Furthermore, it was shown that female practitioners attended funerals to support the family (*P* = .016) and to express their personal grief (*P* = .004).^[35]^ Male practitioners, on the contrary, were more likely to attend funerals to gain public acknowledgment from the patient's relatives (*P* = .036); in addition, they were less likely to regret not attending funerals (*P* = .007).^[35]^ However, there is no study that discovered statistical significance between gender and the rate of funeral attendance. Therefore, it is uncertain whether female practitioners attend funerals more frequently than their male counterparts.

Younger doctors had a more sceptical attitude toward attending a patient's funeral. Zambrano et al^[35]^ discovered that practitioners who have not attended funerals tended to be younger than those who have attended (*P* < .001). MPs who have not attended a patient's funeral disagreed more with the statement that they are more likely to attend a patient's funeral as they become older (*P* < .001).^[35]^ Similarly, 75% of the participants from the Borasino et al^[42]^ study did not change their funeral attendance practice over time. Of those who did, half of them increased their attendance rate, while the other half decreased it.^[42]^

Nevertheless, more recent studies suggested that the overall rate of funeral attendance may have increased over the past few decades. For instance, anonymous surveys distributed in 1996 in an American hospital showed that no pediatric residents and 25% of the fellows attended a patient's funeral.^[40]^ A single-site American study conducted from 2001 to 2004 revealed that 15% to 35% of pediatric trainees have attended a patient's funeral.^[44]^ Further, a study published in 2008, which involved online surveys completed by American pediatric intensivists, revealed that 71% of participants had attended a funeral at least once.^[42]^ In 1988, only 9% of patient funerals were attended by oncologists.^[46]^ Considering that it was likely for oncologists to attend multiple funerals, the actual rate of attendance would be even lower. Indeed, recent studies showed that between 19.1% and 67% of oncologists have attended funerals.^[35,47]^

Years in practice can potentially influence bereavement practice. According to Zambrano et al,^[35]^ practitioners who have not attended their patient's funeral tended to have less years of experience (*P* < .001). As mentioned in the previous paragraph, pediatric fellows were more likely to have attended a patient's funeral, compared to residents.^[40]^ Similar results were obtained from Senthil et al,^[44]^ who found that 35% of pediatric trainees had attended their patient's funeral, as opposed to 1st and 2nd years (15% and 24%, respectively) in the last 12 months. Multiple factors could have contributed to this, including the difference in age, level of responsibility and the degree of rapport formed between the MP and the patient or the family.

The relationship between the MP and the patient or the family was an important factor with regard to funeral attendance. Using 8 imaginary clinical situations, Borasino et al^[43]^ examined several factors that could influence a practitioner's attendance at a patient's funeral: the patient's length of stay, the family's degree of trust toward the MP, and the family's emotional reaction to the patient's death. The degree of the family's trust toward the MP was the only significant facilitator of funeral attendance.^[43]^ About 38.6% of participants reported that they would attend the funeral of their patient when the family trusts them. In comparison, 7.8% claimed that they would attend the funeral of their patient when the family does not trust them (*P* < .001).

### 2.11 Other factors that have been suggested in the literature

Geographic factors may also influence funeral attendance. The physical location of the MP in relationship to the patient or the family may also be a reason for funeral attendance.^[44]^ It was suggested that rural practitioners are more likely to attend patient funerals, as they tend to form closer relationships with their patients.^[7,32]^ However, there is no research that involves a large group of participants and that specifically studies the relationship between the area of practice and funeral attendance.

Previously, practitioners noted that they attended patients’ funerals to gain a better understanding about the patient.^[8,9,19]^ Attendance at a funeral may allow MPs to gain insights as to what the patient was like outside the hospital or clinic and to gain a better understanding of the person they were treating.^[8,9]^ Even general practitioners who often form a long-term relationship with their patients commented about the difficulties of really knowing patients before they started treating them for a particular illness.^[8]^ An emergency physician described funeral attendance as the “transformation of 2-dimensional patient encounters into a 3-dimensional picture.”^[9]^ An oncologist suggested that although such experiences did not instantly transform her into a better doctor, they allowed her to gain considerably more insight about the patient.^[12]^ To date, there is no study that evaluates whether gaining a better understanding of patients is a motivating facilitator for doctors to attend patients’ funerals.

With time, MPs and patients can form a special bond over the course of multiple professional encounters. Some MPs admitted that they often felt a need to say farewell to the patient who had become a friend.^[7,20]^ Pediatric trainees identified “feeling close to the patient or the family” as the most common reason for funeral attendance.^[44]^ Among Israeli oncologists, the rate of funeral attendance elevated from 7.4% for general patients to 28.4% for patients with whom they formed a special bond.^[39]^

### 2.12 Barriers to attendance

#### 2.12.1 MP factors

Time restraint was the most commonly cited and studied barrier in the literature.^[7,15,17,19,20,34,36,37,40,42,44]^ Some, however, criticized this position through editorials and letters, stating that time is an “excuse” for not attending a funeral.^[15]^ In fact, Hood^[20]^ admitted that he realized that time was not a true barrier to funeral attendance. Similarly, the most recent cross-sectional study involving a larger number of participants across several specialties failed to identify time as a barrier to attendance.^[35]^ Despite this data, several cross-sectional studies involving pediatricians and pediatric trainees have identified time restraint as the main barrier to funeral attendance. For instance, 39% of pediatric critical care specialists and 47% of pediatric trainees reported time or logistic difficulties as the main reason why they did not attend their patient's funeral.^[40,42,44]^ Similarly, the results from another Australian study concluded that the majority of the palliative care specialists did not attend funerals due to time restraints.^[36]^ Overall, the evidence appears contradictory and perhaps context specific. Further research needs to be conducted to determine whether time is a true barrier to funeral attendance.

The MPs feared that professional and personal boundaries can be blurred by attending a patient's funeral. Loblay^[32]^ argued that he would only consider attending the funerals of patients with whom he had developed “a close and long-standing relationship,” and that he was even selective in attending his patients’ celebratory events. Colleagues of Peters^[7]^ expressed similar concerns. Zambrano et al^[35]^ discovered that practitioners who have not attended a patient's funeral were more likely to agree with this idea. Such a belief was independently associated with nonattendance (odds ratio 0.458, 95% CI 0.312–0.672, *P* < .001).^[35]^

Previously, Lundberg^[1]^ suggested that MPs fear attending funerals because “they are so scared of what they are trying to prevent” (that is, death). Funeral attendance was emotionally challenging for practitioners, and evoked fear and guilt, with some authors even arguing that regular attendance at funerals accelerated emotional and physical burnout.^[30,31]^ Personal feelings of discomfort or feeling “too emotional” were the second most commonly quoted reason for not attending funerals among pediatric critical care physicians.^[42]^ Similarly, in another study almost half of the pediatric residents feared attending patients’ funerals.^[2]^ In the same study, 40% of participants had felt guilty about a patient's death, which could have caused them to fear attending their patient's funeral. Morris,^[17]^ on the contrary, insisted that funeral attendance prevented professional burnout because attendance was so beneficial for professional learning. This difference suggests that whether funeral attendance is perceived as a cause of burnout depends on the individual practitioner's perception of funeral attendance.

The atmosphere of the working environment also seemed to prevent MPs from attending funerals. This position appeared to be more prominent in certain medical specialties. An emergency physician admitted that his colleagues were surprised to know that he attended patients’ funerals on a regular basis.^[9]^ Srivastava^[12]^ also claimed that MPs were expected adopt a stoic approach, to appear strong and not to discuss a patient's death. The results from Zambrano et al's^[35]^ study suggested that intensive care and surgery are 2 specialties groups that disapproved of funeral attendance. Intensivists and surgeons were less likely to discuss funeral attendance with colleagues compared to palliative care specialists and GPs (*P* < .001). Moreover, surgeons were more likely to believe that their colleagues will disapprove of funeral attendance compared to palliative care specialists (*P* = .004). In contrast, McCabe et al^[45]^ found that 65% of pediatric residents who had attended a patient's funeral had been encouraged to do so by a colleague.

The MPs often perceived a patient's death as their own failure.^[2,27]^ Consequently, they feared that their presence at the funeral might remind the family about the death or be interpreted as intrusive.^[2]^ Indeed, 20% of pediatric critical care physicians thought that the family would deem an MP's actions intrusive or inappropriate if he or she attended the funeral.^[42]^ Moreover, Zambrano et al^[35]^ identified personal discomfort with death as a variable that discouraged an MP from attending the patient's funeral (odds ratio 0.636, 95% CI 0.428–0.945, *P* = .025). In summary, MPs often developed a sense of failure and unease because of a patient's death, which could hinder them from attending patients’ funerals.

#### 2.12.2 Family/patient factors

An MP's attendance at a patient's funeral carried risks of breaching confidentiality. Markowitz^[11]^ warned that if the patient does not want to disclose his or her relationship with the MP, even attending the funeral can break confidentiality. Additionally, some MPs were concerned that the presence of a practitioner could invite clinically inappropriate questions.^[7,34]^ However, Srivastava^[12]^ asserted that clinical questions were not asked by the family, despite this concern. Richardson^[34]^ further claimed that attendance at funerals could create suspicion, as the family would view such attendance as a way for the practitioner to ease his or her guilt regarding the loss of the patient. Indeed, MPs often believed that the family would not welcome their presence at the funeral.^[8,19,20]^ Despite this concern, many practitioners who have attended a patient's funeral illustrated that they were greeted with honor by the family, instead of with suspicion, and anger.^[9,19–21]^ In an educational article, Jain and Jain^[25]^ also claimed that psychiatrists are advised to attend the funeral of patients who committed suicide, as the family appreciates his or her presence, and that attendance is not an admission of guilt. In fact, in a qualitative study that involved the families of deceased children, researchers discovered that families appreciated healthcare professionals’ attendance at the funeral and were disappointed by their absence.^[3]^ Interestingly, Grossman^[30]^ insisted that he did not attend patients’ funerals because he could not “pick and choose” which funeral to attend. Similarly, Richardson^[34]^ warned that attendance might create a sense of discrimination among families if the practitioner did not attend a funeral.

A couple of other patient factors had been studied in the literature. Borasino et al^[43]^ examined if the patient's length of stay in the hospital (24 hours vs 12 days) influences the MP's participation in bereavement practices using eight imaginary scenarios. The results showed that MPs’ involvement in bereavement practices does not change. The rate of making a phone call or scheduling an office appointment with the family were 30% (*P* = .94) and 29% (*P* = .91), respectively whether the patient stayed in the hospital for 24 hours or 12 days. About 32% of participants said that they would send a condolence card for patients who stayed in the hospital for 24 hours. That for patients who stayed in the hospital for 12 days was 33% (*P* = .88). In addition, whether the patient dies as an inpatient or an outpatient did not influence the participants’ rate of involvement in bereavement practices (condolence note, calling the family, home visit, and attending funeral, *P* = .06).^[38]^

### 2.13 Benefits of funeral attendance

#### 2.13.1 Benefits to the family

The MPs attended funerals to assist with the family's grieving process.^[6,11,13,14,16,18–20,36,44]^ Doctors believed that their presence at the funeral is interpreted as a continuation of care for the family, despite the patient's death.^[36]^ Some reports stated that doctors also cared about “the family's well-being” and that they were “happy to be contacted.”^[6]^ In addition, families may doubt the decisions they had made for their loved ones, and consequently experience feelings of guilt; MPs could often ease that sense of guilt by addressing the family's questions and concerns. Data from Senthil et al^[44]^ showed that 40% of pediatric residents attended funerals to support the family, suggesting that support for the family is a commonly perceived benefit of funeral attendance among MPs.

Some MPs believed that they were obliged to support the family or that they were caring for the family as part of their overall care for the patient.^[13,14,18]^ For instance, Chuparkoff^[14]^ described her father's frequent attendance at patients’ funerals as a general practitioner, because he felt “obliged to take care for the entire family.” Such a sense of responsibility was not shared exclusively among general practitioners. A surgeon also stated that he attended funerals because he “feels obliged to support the family.”^[13]^

Clinicians considered funeral attendance as an opportunity to maintain or extend the relationship with the patient's family.^[7,11,16]^ This practice was frequently noted by family physicians or general practitioners who often provided medical care to the entire family.^[7,16]^ Peters^[7]^ described 2 incidences in which he attended the funeral of his patient and was able to maintain a professional relationship with the survivors. On the contrary, he lost contact with one patient's wife when he sent her a condolence letter instead of attending the funeral. This exemplifies how funeral attendance can result in the formation of strong bonds with the family. Similarly, Markowitz^[11]^ believed that funeral attendance is an opportunity to “extend the physician's role” to the family, and he described how he provided further support for family members.

Funeral attendance was suggested as a gesture of respect and love toward the patient and the family.^[16,18,35]^ Irvine^[18]^ suggested that the presence of an MP gives the family a sense that the deceased was a special person to the doctor. This was previously suggested in a letter by Landau,^[16]^ who stated that he attended funerals as a token of love to the family. A multivariable analysis of factors that influence an MP's decision to attend a patient's funeral suggested that “attending funerals to show respect to the family” was the strongest driver of funeral attendance (odds ratio 2.072, 95% CI 1.369–3.136, *P* = .001).^[35]^ Collins-Tracey et al^[6]^ disagreed with this explanation, as their qualitative study showed that very few of their participants attended funerals to show respect to the family. However, their study was limited in terms of the number of participants (11 MPs) and the specialties involved (palliative care and oncology).

#### 2.13.2 Benefits to the MP

Following a patient's death, MPs often experienced grief or a sense of guilt and responsibility.^[19,20,36,44]^ Although MPs could generally confine the bereavement to a professional level, they found it difficult to cope if they encountered many deaths over a short period of time, had a close relationship with the patient, or found that the goals of the treatment had not been met.^[36]^ Irvine^[18]^ and Hood^[20]^ illustrated their experiences at a funeral in which the family comforted them and helped them to escape from their sorrow over the loss of a patient. In fact, attendance at patients’ funerals as a part of self-care was the 2nd strongest facilitator to funeral attendance, with an odds ratio of 1.842 (95% CI 1.171–2.898, *P* = .008).^[35]^

Personal growth was a commonly mentioned benefit of funeral attendance.^[12,13,17]^ Some authors suggested that funeral attendance was a humbling experience that motivated one to be a more considerate doctor.^[12,13]^ Similarly, a medical oncologist previously described funeral attendance as a personally enriching experience that was beneficial in emotional, professional, social, and educational aspects.^[17]^ The author also suggested that MPs can gain interpersonal skills by conversing with the relatives.^[17]^ Despite these seemingly plausible arguments, there is no quantitative data that explores whether this is a commonly perceived benefit among MPs.

### 2.14 Difference in specialties

A literature search identified 13 quantitative studies that explored the rate of funeral attendance by MPs. All of them were conducted in developed countries and employed a physical questionnaire or an online survey as the study material.

General practice could be considered a unique specialty where attendance of a patient's funeral by the general practitioner could be easier to facilitate. As depicted by Peters,^[7]^ frequent encounters with a patient could result in a professional relationship evolving into a friendship. It was also common for general practitioners to form a close relationship with the patient's family.^[7,8]^ Family trust was described as a facilitator to attending a patient's funeral.^[43]^ Zambrano et al^[35]^ found that general practitioners most extensively discussed funeral attendance among themselves (*P* < .001) and were the least likely to disapprove of a colleague's attendance (*P* < .001); both of these factors were associated with a higher rate of funeral attendance. As a result, general practitioners had the highest rate of attendance at 71%.^[35]^

Zambrano et al^[35]^ suggested that 63% of palliative care physicians attended a patient's funeral. This percentage, however, could be an overestimation, possibly due to the high prevalence of female palliative care physicians surveyed (65%). Chau et al^[37]^ presented a funeral attendance rate attendance of 81%; however, this percentage included attendance at either a funeral or a memorial service. Only 16% of American oncologists and palliative care physicians reported that they always or usually attend their patient's funeral.

Studies failed to agree on the rate of oncologists’ attendance at funerals. According to Zambrano et al,^[35]^ where 27 oncologists were involved, the rate was similar to that of palliative care physicians, at 67%. Kusano et al^[47]^ also suggested that only 16 would usually or sometimes attend their patient's funeral. Chau et al^[37]^ which involved 535 oncologists showed that 45.5% of medical oncologists and 35% of radiation oncologists attended a patient's funeral. Also, Chau et al^[37]^ had a higher participation rate at 71%, compared to Kusano et al,^[47]^ at 19.1%. Therefore, one could postulate that the data from Chau et al^[37]^ better reflected the actual practice. Corn et al^[39]^ determined that only 7.4% of Israeli oncologists attended a patient's funeral. However, the survey of this study only offered frequently, occasionally, and never as options for funeral attendance, contrary to Chau et al,^[37]^ who also offered rarely as an additional option. In fact, 40.7% of medical oncologists and 30.6% of radiation oncologists rarely attended a patient's funeral in the study by Chau et al.^[37]^

Other medical specialties are relatively understudied. An anonymous questionnaire discovered that 15% of 109 Irish psychiatrists attended the funeral of patients who had committed suicide.^[48]^ More recent data from Zambrano et al^[35]^ suggests that the rate is 67%, but this data cannot be generalized, as the number of psychiatrists involved in the study was only 12. Funeral attendance by surgeons has been reported at 52% of 68 participants.^[35]^ ICU physicians had the lowest rate of attendance among all of the specialties that were studied in Zambrano et al,^[35]^ at 22% of 62 participants. None of the 114 American emergency physicians in one study had attended a patient's funeral, based on a quantitative research published in 1990.^[41]^

Specific to anesthesia, the best evidence to date can be extrapolated from Borasino et al^[42]^ in which 22 of 204 pediatric critical care specialists were subspecialized in anesthesia, and Zambrano et al,^[35]^ where 5 ICU specialists had a combined specialty in anesthetics. Borasino et al^[42]^ identified the rate at 71%, whilst Zambrano et al^[35]^ claimed it was 22%. Borasino et al's^[42]^ study encompassed a wide range of subspecialties, including anesthetics, cardiology, pulmonary, general pediatrics, palliative care, and others, meaning that the results may reflect pediatricians in general, rather than anesthetists. Anesthetists’ encounters with patients tended to be brief, and it was difficult for them to build a long-term and in-depth relationship with patients and families.^[28]^

## 3 Conclusion

We performed a systematic review to explore funeral attendance as a bereavement practice among MPs, particularly focusing on the factors that influenced MPs’ attendance and the difference in attendance rate between disciplines. We found that attendance at a patient's funeral is a less popular bereavement practice among MPs. Age, gender and years in practice were demographic factors that could potentially influence MPs’ attendance. The degree of rapport and trust between the clinician and the patient or the family were also important determinants of MPs’ attendance at their patient's funeral. Furthermore, the prevalence of attendance at a patient's funeral varied between the limited number of medical specialties that have been studied.

Our study has a few limitations. First, a meta-analysis could not be conducted due to the significant heterogenicity of the methodology and study characteristics of the selected papers. In addition, there is a paucity of quantitative research exploring factors that influence MPs’ involvement in bereavement practice and the rates of the involvement. As a result, parts of this review were written in descriptive manner.

Our study addresses a few gaps in the literature. First, the practice of attending a patient's funeral needs to be studied in other medical specialties, such as in anesthesia. This will help define the characteristics of MPs that influence their attendance at a patient's funeral. In addition, studies disagree on whether time restraint and emotional challenges are true barriers to MPs’ attendance at their patient's funeral. Moreover, there are not enough quantitative studies that explore MPs’ participation in bereavement practices. Future literature of quantitative nature are needed for an in-depth study of the topic, such as meta-analysis of factors that influence MPs’ involvement in bereavement practices especially patient factors including the patient demographics, diagnosis, and the duration for which the MP had known the patient for. Finally, most of the studies identified in this review were conducted in Western countries. Therefore, it is possible that practitioners from non-Western countries may have different views toward bereavement practices. Attendance at a patient's funeral is an important aspect of medicine from which both the treating team and the family can benefit.^[3,12,13,35]^ Future research to explore the attitudes toward, benefits of, and barriers to attending a patient's funeral is therefore still justifiable.

## Author contributions

**Conceptualization:** Kwangtaek Kim, Leonid Churilov, Andrew Huang, Laurence Weinberg.

**Data curation:** Leonid Churilov.

**Formal analysis:** Leonid Churilov.

**Methodology:** Kwangtaek Kim, Leonid Churilov, Laurence Weinberg.

**Project administration:** Laurence Weinberg.

**Resources:** Kwangtaek Kim, Laurence Weinberg.

**Supervision:** Leonid Churilov, Andrew Huang, Laurence Weinberg.

**Validation:** Kwangtaek Kim, Andrew Huang, Laurence Weinberg.

**Writing – original draft:** Kwangtaek Kim.

**Writing – review & editing:** Kwangtaek Kim, Leonid Churilov, Andrew Huang, Laurence Weinberg.

Kwangtaek Kim orcid: 0000-0002-1492-4971.

## Supplementary Material

Supplemental Digital Content
