# Supplementary material for: Bereavement practices employed by hospitals and medical practitioners toward attending funeral of patients: A systematic review
Source: Medicine (Baltimore). 2019 Sep 6;98(36):e16692. doi: 10.1097/MD.0000000000016692 (PMC6739025; doi:10.1097/MD.0000000000016692)
Supplement: Supplemental Digital Content [file medi-98-e16692-s001.docx]

**Appendix**

**Medline (Ovid) search history**

1. Exp Professional-Family Relations/

2. Exp Professional-Patient Relations/

3. Exp FUNERAL RITES

4. Exp Physician-Patient Relations/

5. 1 or 2 or 4

6. 3 and 5

7. Limit 6 to English

8. Limit 7 to nursing journals

9. 7 not 8

**EMBASE search history**

1. Exp health personnel attitude/ or exp attitude to death/ or exp physician attitude/

2. Exp doctor patient relation/

3. exp posthumous care/

4. 1 or 2

5. 3 and 4

6. Limit 5 to English
